# First Nations Peoples’ Participation in the Development of Population-Wide Food and Nutrition Policy in Australia: A Political Economy and Cultural Safety Analysis

**DOI:** 10.34172/ijhpm.2020.175

**Published:** 2020-09-26

**Authors:** Jennifer Browne, Michelle Gilmore, Mark Lock, Kathryn Backholer

**Affiliations:** ^1^Global Obesity Centre, Institute for Health Transformation, Deakin University, Geelong, VIC, Australia.; ^2^School of Health and Social Development, Deakin University, Geelong, VIC, Australia.; ^3^Committix Pty Ltd., Newcastle, NSW, Australia.

**Keywords:** Indigenous health, Aboriginal Health, Food Policy, Nutrition Policy, Cultural Safety, Australia

## Abstract

**Background:** Healthy and sustainable food systems underpin the well-being of Indigenous peoples. Increasingly governments are taking action to improve diets via population-wide policies. The United Nations Declaration on the Rights of Indigenous People states that Indigenous peoples have the right to participate in all decisions that affect them. We analysed Australian national food and nutrition policy processes to determine: (i) the participation of Aboriginal organisations, (ii) the issues raised in Aboriginal organisations’ policy submissions, and (iii) the extent to which Aboriginal organisations’ recommendations were addressed in final policy documents.

**Methods:** Political economy and cultural safety lenses informed the study design. We analysed publicly-available documents for Australian population-wide food and nutrition policy consultations occurring 2008-2018. Data sources were policy documents, committee reports, terms of reference and consultation submissions. The submissions made by Aboriginal organisations were thematically analysed and key policy recommendations extracted. We examined the extent to which key recommendations made by Aboriginal organisations were included in the subsequent policy documents.

**Results:** Five food and nutrition policy processes received submissions from Aboriginal organisations. Key themes centred on self-determination, culturally-appropriate approaches to health, and the need to address food insecurity and social determinants of health. These messages were underrepresented in final policy documents, and Aboriginal people were not included in any committees overseeing policy development processes.

**Conclusion:** This analysis suggests that very few Aboriginal organisations have participated in Australian population-wide food and nutrition policy processes and that these policy development processes are culturally unsafe. In order to operationalise First Nations peoples’ right to self-determination, alternative mechanisms are required to redress the power imbalances preventing the full participation of Aboriginal and Torres Strait Islander peoples in population-wide food and nutrition policy decisions. This means reflecting on deeply embedded institutional structures and the normative assumptions upon which they rest.

## Background

 Before colonisation, First Nations peoples worldwide were the custodians of sustainable food systems which maintained human health and protected biodiverse ecosystems for millennia.^1^ For many First Nations peoples food is more than a source of energy and nutrients; it is inculcated in cultural identity, land, family and history, as well as physical, social, emotional and spiritual well-being.^2,3^ European colonisation severely disrupted and marginalised the cultural practices and food systems of First Nations peoples around the world, manifesting in the social, economic and health inequities that persist today.^1-3^ Nevertheless, First Nations peoples have survived and continue to hold cultural, environmental and food system knowledge, which is a valuable resource, not only for the health of First Nations peoples, but also for the sustainable development of the broader industrialised world.^4,5^

Aboriginal and Torres Strait Islander peoples are the traditional owners of Australia with a history of over 65000 years, representing the world’s longest surviving continuous culture.^6,7^ In this paper we respectfully use the term ‘Aboriginal’ to describe First Nations peoples of Australia, acknowledging that these comprise many different nations, language groups, geographic areas, cultural and kinship systems. Aboriginal people have developed healthy and sustainable food systems based on transgenerational knowledge of seasonal food sources and preparation methods.^3^ Aboriginal people hunted and gathered, practiced agriculture and aquaculture, and made sophisticated technological innovations to procure and process food.^8,9^ As a result the precolonial diets of Aboriginal people were derived from a wide variety of plant and animal foods, high in protein, fibre and micronutrients and low in fat, sugar, and salt.^10,11^

Precolonial Aboriginal society was healthy, self-determined and free from diet-related chronic disease.^3,11^ This changed with European invasion and colonisation of Australia because Aboriginal people were dispossessed from their homelands and denied access to their cultural knowledge and food systems.^3^ Introduced species and Western agricultural practices caused environmental degradation further reducing availability of traditional food sources.^9^ Traditional, nutrient-dense diets were first replaced by government-controlled rations of flour, rice, sugar, tea and, occasionally, poor-quality meat; then by imposed colonial-style cooking during the policy of assimilation, which aimed to extinguish all traces of Aboriginal cultures.^12^ The presence of malnutrition was often used to justify the forced removal of Aboriginal children from their families, further disrupting the passage of traditional food knowledge through the generations.^3^ Thus, changes to food and nutrition were both an instrument and an outcome of colonisation.

Colonisation and industrialisation of traditional food systems have dramatically changed the diets of First Nations peoples internationally.^1^ Like other First Peoples, Aboriginal Australians have undergone a nutrition transition characterised by a rapid Westernisation of dietary patterns and sharp increase in prevalence of obesity and chronic disease.^13^ Dietary factors and high body mass are each responsible for approximately 15% of the gap in health outcomes between Aboriginal and non-Aboriginal Australians.^14^ Furthermore, more than 1 in 5 (22%) Aboriginal people experience food insecurity, with the prevalence even higher (31%) in remote Australia.^15^ The social determinants of health, including education, employment, income and housing, all contribute to the food and nutrition inequity experienced by Aboriginal people.^16^ These determinants are underscored and amplified by the historical and ongoing processes of colonisation, dispossession and disempowerment as well as interpersonal and institutional racism.^17,18^

The factors underpinning Aboriginal food and nutrition are ultimately political. First Nations academics argue that cultural imperialism, institutional failure and unequal distribution of power and opportunities for political participation have produced policies which contribute to health inequity for Aboriginal people.^19-21^ Moreover, the absence of a treaty or constitutional arrangements guaranteeing Aboriginal people a political ‘voice’ to public institutions sets Australia apart from similar Western colonised nations when it comes to the rights of First Nations peoples.^19^ The United Nations (Article 18) has affirmed that:

*“Indigenous peoples have the right to participate in decision-making in matters which would affect their rights, through representatives chosen by themselves in accordance with their own procedures.”* ^2 2^

In this article, we examine the extent to which Aboriginal people, through their representative organisations, have had a voice in developing population-wide policies related to healthy and sustainable food systems in Australia. In the Australian context, Aboriginal community-controlled organisations, which are governed by and accountable to their local Aboriginal communities, frequently represent Aboriginal peoples in policy debates regarding matters that affect them.^23^ Below, we provide a brief overview of the political context of Aboriginal affairs and food and nutrition policy-making. We then introduce our intersectional approach to policy analysis combining two theoretical perspectives which we apply in this examination of food and nutrition policy-making in Australia.

###  Political and Policy Context

The Commonwealth of Australia is a federation comprising 6 states and 2 territories, established in 1901. The Federal Government has only had the power to develop policy for Aboriginal people since 1967, following a landmark referendum which enabled Aboriginal people to be officially counted in the Australian population.^24^ This occurred during a time of civil rights activism, which also saw the establishment of the first Aboriginal community-controlled organisations, which were “an important institutional manifestation of the politics of self-determination.”^24^ From the 1970s, self-determination was part of the Government policy approach to Aboriginal affairs, with Federal funding provided to Aboriginal organisations and the establishment, in 1990, of a national representative and administrative body, the Aboriginal and Torres Strait Islander Commission (ATSIC).^25^ The conservative opposition party voted against the creation of ATSIC and when they came to power, in 1996, had a fractious relationship with Aboriginal leaders. The abolition of ATSIC, in 2004, marked the end of the era of self-determination in Aboriginal affairs policy.^25,26^

There has been a long history of Aboriginal involvement in food and nutrition policy. For example, when ATSIC was in operation, one of its responsibilities was the Community Housing and Infrastructure Program, which included guidelines and standards for housing design to enable, amongst other things, adequate food storage and preparation.^27^ However, there has only been one dedicated national food and nutrition policy, the National Aboriginal and Torres Strait Islander Nutrition Strategy and Action Plan 2000-2010 (NATSINSAP).^27^ NATSINSAP was developed, following broad Aboriginal community consultation and oversight, as a component of *Eat Well Australia*, Australia’s population-wide food and nutrition strategy. Since NATSINSAP and *Eat Well Australia* expired in 2010, there has been no comprehensive national nutrition policy for either the general Australian population or for Aboriginal peoples.

In 2007, the United Nations adopted the Declaration on the Rights of Indigenous Peoples, which included the right to self-determination.^22^ Australia, along with New Zealand, Canada and the United States, initially voted against the declaration; however, eventually supported it following the election of a new Labor (social democratic) Federal Government. A watershed moment in Australian politics occurred in February 2008, when the Prime Minister delivered a Parliamentary apology to the Aboriginal people, known as the * Stolen Generations,* who, as children, were forcibly removed from their families by previous Governments under the policy of assimilation. The same year, following an Aboriginal-led, rights-based advocacy campaign, the Prime Minister and Opposition leader made a bipartisan commitment to “close the gaps” in health status and life expectancy between Aboriginal and non-Aboriginal Australians, with the full participation of Aboriginal people and their representative bodies.

Over 10 years have passed since these commitments were made and the health gap persists. The *Closing the Gap* policy framework has been criticised for being based on a deficits discourse, which defines success as achieving statistical parity with the non-Aboriginal ‘norm,’ rather than outcomes defined by Aboriginal people.^28^ This is likely because it was developed by the Council of Australian Governments without substantive formal Aboriginal participation in the policy process.^29^ Furthermore, with the exception of a strategy for remote food stores, food and nutrition were almost completely absent from the Closing the Gap policy agenda.^30^ By contrast, the 2013 National Aboriginal and Torres Strait Islander Health Plan was generally welcomed by Aboriginal leaders as it was developed following extensive engagement with Aboriginal organisations and communities via written submissions and consultation forums. It emphasised the cultural strengths of Aboriginal people as central to health policy and the need to address racism.^31^ The Health Plan also included significant food security and nutrition elements; however, the Federal Labor Government lost the election soon after the Health Plan was launched and, as a result, it remains largely unimplemented.^32^ Nonetheless, Aboriginal and Torres Strait Islander communities and organisations have continued to advocate for its implementation and for more meaningful engagement in the policy process. In 2019, the partnership agreement between the Council of Australian Governments and a coalition of Aboriginal community-controlled peak organisations recalibrated the nature of policy development to ensure that policies directly affecting Aboriginal people are based on shared decision-making and consider cultural safety and self-determination.^33^ This culminated, in 2020, in the publication of a revised set of Closing the Gap targets which, for the first time, were developed in partnership with Aboriginal peak organisations.^34^

Promoting healthy and sustainable food systems and improving population nutrition requires a comprehensive, multisector policy response. From an equity perspective, targeted food and nutrition strategies should be complemented by population-wide policies, both of which must meet the needs of the Aboriginal population as determined by them.^35,36^ The prominence of food and nutrition on the Federal Government’s Aboriginal health policy agenda has varied over time for a number of reasons.^37^ Furthermore, 80% of Government funding for Aboriginal people is spent through universal, population-wide programs and services.^38^ It is these population-wide policy processes that are the focus of the current analysis. Food and nutrition policy development in Australia involves a varying degree of public consultation. Inviting written submissions is a common strategy used to engage stakeholders in the policy process. Following a public consultation, stakeholder submissions are usually published on publicly-available Government websites.

###  Conceptual-Methodological Approach

The multiple and intersecting concerns related to policy development and Aboriginal peoples require appropriate methodologies. As a team comprising both Aboriginal and non-Aboriginal researchers we drew on Durie’s concept of ‘research at the interface’ between Indigenous and non-Indigenous knowledge systems.^39^ To operationalise this idea, we combined political economy and cultural safety perspectives to examine Aboriginal organisations’ participation in population-level food and nutrition policy-making processes.

There are calls for nutrition advocates and policy-makers to apply political economy analyses to improve policy-making for healthy and sustainable food systems.^40,41^ However, political economy theories have been underutilised in the context of Aboriginal food and nutrition. The international food and data sovereignty movements highlight the political, economic and cultural nature of First Nations peoples’ aspirations for food system transformation, and the need for appropriate evaluation indicators and data governance.^1,2,4^ Political economy analysis is concerned with the interaction of political and economic processes and the distribution of power and resources between different groups in society.^42^ This includes examination of the roles of institutions, incentives, ideas, interest groups and power relations in influencing policy development.^40^

Key to this approach are questions of signification (who makes the rules?), domination (who allocates the resources?), legitimation (what is legitimate knowledge?) and interpretation (how we understand and frame the world?)^43^ In theory, an individual actor or interest group has the freedom to contribute ideas in policy debates, however their power to influence decisions is shaped by the historical, social, cultural, political and economic properties of society. Policy-making has embedded and invisible institutional structures, rules and resources that are drawn-on throughout the policy process, which may either enable or constrain the participation of certain groups.^43^ In order to harness these constructs, our analysis was informed by Hall’s political economy framework, which posits that policy processes are determined by actors’ interests and ideas and mediated through institutions.^37,41,44^

Political economy frameworks do not explicitly account for cultural identity. We refer above to the cultural destruction of Aboriginal food systems and the enduring impacts of the policies imposed, without consultation, on Aboriginal Australians by the colonial governments. The food sovereignty movement seeks to reframe relationships between Western governments and First Nations Peoples through, for example, consideration of cultural identity and participation in policy-making processe.^1,2,4^ Additionally, there has been a cultural policy reform agenda in Australia, emphasising the cultural strengths of Aboriginal peoples as central to all health policy.^45-47^ When developing food and nutrition policy in Australia it is, therefore, essential to recognise the influences of historical trauma and ongoing colonisation, dominant culture, white privilege and power, and both overt and institutional racism, which serve to devalue the cultural strengths of Aboriginal people.

The concept of “cultural safety” – championed by Māori nurse Irihapeti Ramsden^48^ – has increased in prominence in Australian health policy as a lens for analysing the power relations between health personnel and the people they serve.^49,50^ Cultural safety is concerned with ‘reflexivity, dialogue, reducing power differences, decolonization and regardful care,’ concepts that challenge the institutional norms of public health policy-making.^48,51^ A key challenge for policy-makers is to reflect on how the policy-making process can – unintentionally – embed institutional racism,^51-54^ especially with legislation that renders Aboriginal people ‘legally invisible.’^20,55^ These challenges are further amplified in universal, population-wide policy processes where Aboriginal people are not a key focus. Therefore, aligning political economy (interests, ideas and institutions) and cultural safety (culture, identity and power) theories provides for a unique methodological analysis at the cultural interface.

This study applied political economy and cultural safety lenses to understand how Aboriginal issues have been included and represented in national population-wide food and nutrition policy processes that took place in Australia between 2008 and 2018. We were specifically interested in: (*i*) the participation of Aboriginal organisations in these policy processes; (*ii*) the common issues in the submissions made by Aboriginal organisations; and (*iii*) the extent to which key issues raised in Aboriginal organisations’ submissions were addressed in final policy documents.

## Key Messages

**Implications for policy makers**

- The codesign of population-wide food and nutrition policy-making processes could better enable Aboriginal peoples’ right to self-determination through improved participation and cultural safety.
- Support must be provided to Aboriginal organisations to increase participation in population-wide food and nutrition policy processes. This may be achieved by:
  - Expanding the scope and range of consultation mechanisms to maximise the participation of Aboriginal organisations in food and nutrition policy processes (eg, Community forums in addition to written submissions).
  - Mandate Aboriginal peoples’ membership on all food and nutrition policy development committees.
  - Implement the recommendations made within the Uluru Statement from the Heart, including a constitutionally enshrined Aboriginal voice to Parliament.
  - Ensure adequate and sustainable funding to Aboriginal organisations, that is not burdened by excessive administrative and accreditation requirements, to increase capacity to participate in policy processes.
- Cultural safety needs to be embedded in the institutional design of policy participation processes.

**Implications for public**

Self-determination is a right of Indigenous Peoples that needs to be embedded in food and nutrition policy, strategy and action. Our findings indicate existing national-level, population-wide food and nutrition policy consultation processes systematically disadvantage Aboriginal and Torres Strait Islander Australians. With regard to population-wide food and nutrition policy, Australia is not meeting its obligations under the United National Declaration on the Rights of Indigenous Peoples.

## Methods

###  Scope and Setting

A qualitative policy document analysis was used to examine the inclusion of Aboriginal organisations’ issues in Australian food and nutrition policy processes. Policy analysis is a field of research which can enable understanding of how and why policy proposals are enacted (or rejected) and the values, ideas, interests and institutional contexts underpinning government decisions.^56^ Our analysis of food and nutrition policy-making in Australia spanned the years 2008-2018. The year 2008 was selected as a starting point for this analysis as it marked the commencement of a new Federal Labor Government in Australia, who brought a renewed policy focus on both Aboriginal health and preventative health (including food and nutrition; see background). This period coincides with growing momentum for asserting First Peoples’ rights to equitable health and self-determination both internationally and in Australia.^22,57,58^ Importantly, this timeframe covers the 10-year period following the United Nations Declaration on the Rights of Indigenous Peoples and the commitment by Australian governments to close the life expectancy gap between its First Peoples and non-Indigenous Australians; therefore, this analysis will interrogate the extent to which the Australian government has met these commitments with respect to population-wide food and nutrition policy. This timeline selection also aligns with recommendations that timeframes of at least 10 years are required to adequately study policy processes.^59^ Although both Commonwealth (national) and state/territory governments have responsibilities in food and nutrition policy and Aboriginal affairs in Australia, our analysis focussed on population-wide food and nutrition policy-making at the national level only.

###  Data Collection

Data sources were national (*i*) policy documents, (*ii*) committee reports and committee terms of reference, and (*iii*) stakeholder submissions concerning food and nutrition policy, all of which are considered to be relevant to policy development processes. Documents were collected from publicly available government websites. Relevant policies were initially identified by reviewing the *Scoping study to inform development of a National Nutrition Policy for Australia,*^60^ which provides a detailed timeline of food and nutrition policy developments until 2013. Additional policy processes were identified through searching the Commonwealth Department of Health consultations page (consultations.health.gov.au) and the Parliament of Australia committees and inquiries page (https://www.aph.gov.au/Parliamentary_Business/Committees) using the search terms “food,” “nutrition,” “preventative health” and “obesity.” To be included in the initial analysis, policy development processes had to meet the following criteria:

1. Commenced between January 1, 2008 and December 31, 2018
2. Focused on government policy at the national level
3. Focused on population-level policy development in one of the following areas:
  - food supply/security
  - diet and nutrition
  - obesity prevention
  - breastfeeding or
  - preventative health (as this may include nutrition)
4. Included public consultation with stakeholder submissions published online.

Policies which specifically targeted the Aboriginal and/or Torres Strait Islander population were excluded as were policies that were only relevant to a particular State or Territory context. For the purpose of this analysis an Aboriginal organisation was defined as being controlled and operated by Aboriginal and/or Torres Strait Islander people and/or governed by an Aboriginal Board and formed to pursue agreed objectives that align with Aboriginal values and beliefs.^61^

###  Data Management and Analysis

 Data analysis was guided by political economy and cultural safety lenses. According to the 3 key objectives for this study, data were analysed in 3 distinct stages.

####  Stage 1. Extent of Aboriginal Organisation Participation in Policy Process

 Documents were arranged into a policy timeline to convey institutional time and trajectory. A data extraction template was developed in Microsoft Excel (Version 15.24, 2016). The following details were extracted from the corresponding website and supporting documents of each policy episode (where relevant): governance over policy process (Government department and/or agency responsible, political party in power, government oversight), year policy process commenced, year final policy document was published, policy goal, objectives/terms of reference, consultation process, relevant consultation documents, total number of consultation submissions received, number of consultation submissions received from organisations (as opposed to individuals), number of submissions from Aboriginal organisations and other processes (if any) employed to consult with Aboriginal stakeholders. Committee terms of reference were searched for key words (Aboriginal, Torres Strait Islander, Indigenous) and committee members overseeing the policy development processes were examined for Aboriginal membership by searching the members’ online profiles to detect whether committee members identified as Aboriginal or Torres Strait Islander or represented Aboriginal organisations. The presence or absence of Aboriginal organisations’ submissions to each policy process was identified as well as the proportion of total organisational submissions made by Aboriginal organisations. All policy processes that included at least one publicly-available written submission from an Aboriginal organisation proceeded to the next stage of analysis.

####  Stage 2: Common Themes in Aboriginal Organisations’ Consultation Submissions 

In stage 2, a combination of thematic analysis^62^ and content analysis^63^ was used to examine Aboriginal organisations’ perspectives regarding population-wide national food and nutrition policy within relevant consultation submissions. A second data extraction template was developed to record summary details for each policy consultation processes in which at least 1 Aboriginal organisation participated. The following details were extracted from each submission made by an Aboriginal organisation: the type of organisation, the sector represented, jurisdiction, prominent issues and recommendations.

To synthesise Aboriginal organisations’ priorities across the range of consultation submissions for all national food and nutrition policy processes, the most prominent issues and recommendations were identified using thematic analysis. Thematic analysis involves applying codes, sorting and organising data into related themes. Submissions were uploaded into NVivo analysis software (QSR International) and grouped based on the associated policy process. Consistent with recommended procedures for thematic analysis, the entire dataset was thoroughly appraised for initial observations of any recurring themes.^64^ During a second appraisal of the data, key food and nutrition issues, policy recommendations, concerns about food/nutrition policy actions and other concerns about the policy process were identified. As each idea or recommendation was observed, a code was applied. For example, a ‘food security’ code was applied to a stakeholder recommendation for ‘improved access to fresh food.’ Codes were developed iteratively as each new submission was analysed then, as patterns were identified, codes were grouped into categories, from which higher-order themes were created.^62^ Once themes were derived, content analysis was applied across all submissions to determine which issues and recommendations were most frequently raised by Aboriginal organisations.^63^

####  Stage 3. Extent of Adoption of Aboriginal Organisation’ Issues in Final Policy Documents

 The key Aboriginal organisations’ priorities and recommendations, identified during stage 2, were extracted into a spreadsheet and compared with the relevant final policy document. This included both reports of the committee overseeing the consultation process, which made recommendations to government, and the final government-endorsed policy documents. These documents were deductively analysed to identify the extent to which key food and nutrition issues and recommendations made by Aboriginal organisations were acknowledged and/or included. Key themes (identified in stage 2) were extracted into a table and cross-checked against committee recommendations and government actions in each policy/strategy document. Including both types of outcome document enabled more detailed analysis of whether Aboriginal perspectives had been considered and, where relevant, the point at which Aboriginal organisations’ recommendations appeared to fall off the policy agenda. A rating was applied (eg, action, partial action, deferred, acknowledged, or no action) to each theme according to the degree of committee/government support for this policy priority. Ratings were discussed between a minimum of 2 investigators until consensus was reached.

## Results

Between January 2008 and December 2018, seventeen national food and nutrition-related policy processes were identified. Eight (between 2008-2012) were identified in the National Nutrition Policy Scoping Study,^60^ and the remaining 9 (between 2014-2018) were retrieved from government website searches. Stakeholder submissions were not publicly available for 8 policy processes. Nine had publicly-available submissions and were included in our stage one analysis. Five national food and nutrition policy processes were included in stages 2 and 3 of our analysis (Figure). Details of included policy processes are provided in Tables 1 and 2.

**Figure:**
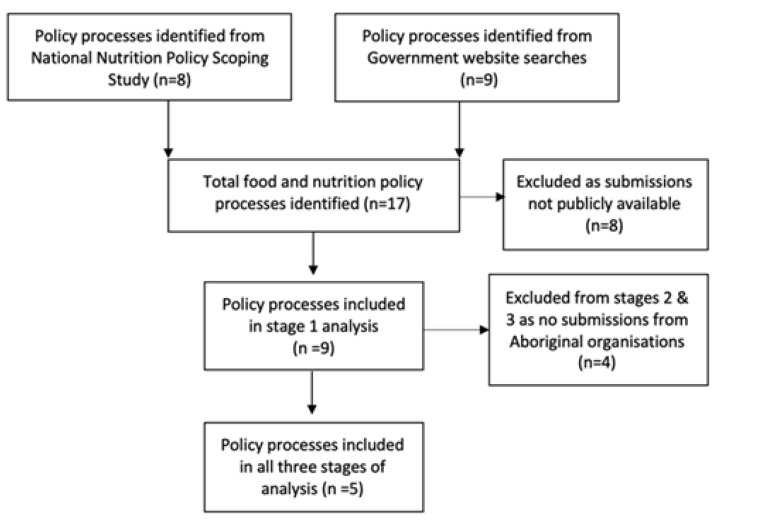
Policy Processes Included/Excluded in the Analysis.

**Table 1:** Aboriginal Organisation Participation in Food Policy Submission Processes

**Policy Process**	**Year Commenced**	**Total Written Submissions**	**Total Submissions From Organisations**	**Submissions From Aboriginal Organisations (% Of Total Organisational Submissions)**	**Total Additional Consultation Events**	**Additional Aboriginal Specific Consultation Events**
NPHS	2008	375	333	5 (1.50%)	39 Public meetings	2 Public meetings
WIU	2008	122	122	1 (0.82%)	16 Public hearings	1 Public hearing
Labelling logic: review of food labelling law and policy	2010	815	212	0	12 Public meetings	0
NFP 2 processes: 1. IP 2. GP	2011	633	IP: 221 GP: 282	IP: 0 GP: 3 (1.06%)	IP: 19 round tables GP: 28 public meetings and 7 round tables	IP: 0 GP: 0
ADG	2011	153	78	1 (1.28%)	0	0
Infant feeding guidelines for health workers	2011	89	46	0	0	0
Country of origin food labelling inquiry	2014	53	39	0	7 public hearings	0
Healthy food partnership voluntary reformulation targets	2018	31	28	0	0	0
Inquiry in the obesity epidemic	2018	151	118	1 (0.85%)	~60 public hearings	0

Abbreviations: NPHS, National Preventative Health Strategy; WIU, Weighing it Up; NFP, National Food Plan; ADG, Australian Dietary Guidelines; IP, Issues paper; GP, Green paper.

**Table 2:** Governance of Food and Nutrition Policy Processes

**Policy Process**	**Political Party in Power**	**Agency Responsible**	**Committee**	**Oversight**	**Total (Aboriginal) Committee Members**
NPHS	Labor	Department of Health and Ageing	National Preventative Health Taskforce	Government	9 (0)
WIU	Labor	Parliament of Australia	House of Representatives Standing Committee on Health and Ageing inquiry	Parliament (House of Representatives)	12 (0)
Labelling Logic: Review of Food Labelling Law and Policy	Labor	Australia and New Zealand Food Regulation Ministerial Council	Independent Expert Panel	Government	5 (0)
NFP	Labor	Department of Agriculture, Forestry and Fisheries	National Food Plan Taskforce	Government	N/A
ADG	Labor	National Health and Medical Research Council	Dietary Guidelines Working Committee	Statutory	13 (0)
Infant feeding Guidelines for Health Workers	Labor	National Health and Medical Research Council	Infant Feeding Sub Committee of the Dietary Guidelines Working Committee	Statutory	4 (0)
Country of Origin Food Labelling Inquiry	Liberal/ National Coalition	Parliament of Australia	House of Representatives Standing Committee on Agriculture and Industry	Parliament (House of Representatives)	10 (0)
Healthy Food Partnership Voluntary Reformulation Targets	Liberal/ National Coalition	Department of Health	Healthy Food Partnership’s Reformulation Working Group	Government	15 (0)
IOE in Australia	Liberal/ National Coalition	Parliament of Australia	Senate Select Committee into the Obesity Epidemic in Australia	Parliament (Senate)	7 (0)
				Total	75 (0)

Abbreviations: NPHS, National Preventative Health Strategy; WIU, Weighing it Up; NFP, National Food Plan; ADG, Australian Dietary Guidelines; IOE, Inquiry into the Obesity Epidemic.

###  Stage 1: Aboriginal Organisations’ Participation in Food and Nutrition Policy Processes

Of the 9 food and nutrition policy processes included in this analysis, 5 included at least 1 written submission from an Aboriginal organisation (55%; see Table 1). Submissions without Aboriginal organisational input into the consultation are also listed in Table 1. Aboriginal organisational input was identified in 5 policy processes: the National Preventative Health Strategy (NPHS; n = 5 Aboriginal organisations), Weighing it up: Obesity in Australia inquiry (WIU; n = 1), the National Food Plan (NFP; n = 3), the Australian Dietary Guidelines (ADG; n = 1), and the Senate Select Committee Inquiry into the Obesity Epidemic in Australia (IOE; n = 1). This resulted in a total of 11 consultation documents for thematic analysis (stage 2). In addition to stakeholder submissions, we noted that several alternative consultation mechanisms were conducted for 6 of the policy processes. These included public meetings, round tables and public hearings; however, these were not consistent across the policy episodes and the level of involvement from Aboriginal organisations was unclear. Only 2 policies (NPHS and WIU) included Aboriginal-specific consultation meetings (Table 1).

Two of these policy processes were parliamentary inquiries (WIU and IOE), 2 were Government strategy development processes (NPHS, NFP) and 1 was a National Health and Medical Research Council guideline development process (ADG). Six of the 9 policy processes with written submissions available occurred under a Labor (social democratic) government and 3 under a Liberal-National (conservative) coalition government. Four of the 5 policy processes that included a submission from an Aboriginal organisation occurred between 2008 and 2011 under the Rudd/Gillard Labor Government and the fifth, a senate inquiry, occurred during the Turnbull Coalition Government (see Table 2).

The terms of reference for the committees (n = 8) overseeing food and nutrition policy processes revealed that of 75 committee members, none were identifiable from their public profiles as First Nations Peoples or representatives of Aboriginal organisations. In most cases, committee members were ‘appointed’ through expert committees or politicians in the case of parliamentary inquiries. Each committee had oversight from the sponsoring organisation, with 4 committees having government oversight, 2 with oversight by statutory (administratively separate from governments but funded by them) organisations, and 3 committees were directly overseen by parliamentarians (Table 2).

 The majority of Aboriginal submissions (n = 7) were from the Aboriginal Community-Controlled Health sector (ACCHS). This included 4 Aboriginal health peak bodies and 1 Aboriginal health service. The remaining 4 submissions were from an Aboriginal health professional association, an Aboriginal-led advocacy coalition, an Aboriginal land council and an Aboriginal economic development organisation. One of the peak bodies made submissions to 4 of the policy processes. The most recent parliamentary inquiry (IOE) received a combined submission from Aboriginal health peak bodies.

###  Key Themes Raised in Aboriginal Organisations’ Submissions

Table 3 presents a summary of the 8 key themes identified in the analysis of submissions from Aboriginal organisations. These were grouped into 3 overarching categories: (*i*) improve healthy food access and promotion, (*ii*) address the social determinants of nutrition, and (*iii*) strengthen governance of public health policy. The most common issues raised were: food insecurity (9/11), inaction on social determinants (8/11), multi-sectoral collaboration (9/11), and – almost unanimously (10/11) – the cultural appropriateness of interventions and services and self-determination. These are elaborated below using illustrative excerpts from submissions.

**Table 3:** Key Themes Raised in Aboriginal Organisations’ Submissions*

**Key Issues Raised in Submissions**	**2008**						**2011**				**2018**	**Total (n/11)**
	**1a**	**1b**	**1c**	**1d**	**1e**	**2a**	**3a**	**3b**	**3c**	**4a**	**5a**	
**Improve Healthy Food Access and Promotion**												
Food insecurity		Y	Y	Y		Y	Y	Y	Y	Y	Y	9
Capacity building for health promotion	Y	Y	Y	Y					Y	Y	Y	6
Food subsidies		Y	Y	Y		Y			Y		Y	5
Industry regulation		Y	Y							Y	Y	4
**Address the Social Determinants of Nutrition**												
Culturally-appropriate programs/ services	Y	Y	Y	Y	Y	Y		Y	Y	Y	Y	10
Inaction on social determinants		Y	Y	Y	Y	Y			Y	Y	Y	8
**Strengthen Governance of Food and Nutrition Policy**												
Self-determination	Y	Y	Y	Y		Y	Y	Y	Y	Y	Y	10
Multi-sectoral collaboration	Y	Y	Y	Y		Y	Y	Y	Y		Y	9

* The top rows of list the de-identified submitting organisations and the year of the submission. The final column shows the frequency of each issue raised in submissions.

###  Improve Healthy Food Access and Promotion

The recurrent issues raised in submissions regarding healthy food access and promotion, in descending order of frequency, were: food insecurity, capacity building, food subsidies and industry regulation. Limited access to affordable, nutritious food, particularly in remote areas was a key theme throughout the 5 policy processes. To highlight this point, submitting organisations cited statistics on the price differential of a standard basket of food between capital cities and remote Aboriginal communities. Food subsidies were frequently recommended to improve food access in remote areas. For example one submission recommended Government reduce the cost of a healthy food basket for Aboriginal families to “ *less than 25% of their available income*” within 10 years (NPHS 1d). Another organisation recommended:

“ *Food subsidies should be highest for fruit and vegetables (given their relatively high cost and nutritious value) but should also be extended to other food staples” *(NPHS, 1c).

Aboriginal organisations provided a range of suggestions for population-wide regulatory measures to reduce consumption of unhealthy foods. One submission *“strongly endorses a total ban on the advertising of junk food”* (NPHS, 1c). Food reformulation, including “ *bans on the use of trans fats, reduction of allowable salt levels”* (NPHS, 1c) were also recommended, as well as *“health-related taxes on sugary drinks and on ‘junk’ food”* (IOE, 5a). This organisation recommended using tax revenue to subsidise healthy foods.

Aboriginal peak bodies opined that community-controlled health organisations frequently delivered effective, culturally-appropriate food and nutrition programs for Aboriginal communities, however lacked the long-term, funding and workforce capacity to sustain program delivery. They called on Government to “ *build health promotion capacity in ACCHSs” *(NPHS, 1c). Suggestions for doing this included nutrition training for the Aboriginal health workforce and developing culturally relevant health promotion *“resources which support healthy eating” *(IOE, 5a).

###  Address the Social Determinants of Nutrition

The majority of Aboriginal organisations were critical of Government inaction on the social determinant of health. They highlighted that ‘behavioural’ approaches to nutrition promotion were favoured rather than “ *addressing structural and environmental changes to improve nutrition status” *(NFP, 3c). The individual risk factor approach was characterised as a Western paradigm, whereas Aboriginal models of health were described as comprehensive and holistic. Stakeholders urged cross-sector collaboration to improve social determinants such as education, employment and housing, and also emphasised * “racism, history, oppression and the ongoing impacts of dispossession” *(NPHS, 1b). One organisation stressed:

*“long-term efforts will be required for the creation of living environments that make healthy choices the easiest ones” *(IOE, 5a).

 One of the most common determinants raised by Aboriginal organisations was the ineffectiveness of mainstream (non-Aboriginal) health services. Submitters called for provision of culturally-appropriate comprehensive primary healthcare and a culturally competent workforce. Aboriginal organisations almost unanimously recommended:

“ *A commitment at all levels of government in terms of funding, policy development, and support for the implementation of culturally-appropriate programs and services” *(IOE, 5a).

###  Strengthen Governance of Food and Nutrition Policy

Aboriginal stakeholders advocated a *“coordinated, whole-of-society approach” *(IOE, 5a) to addressing food and nutrition issues. The importance of collaboration across agencies and across sectors was highlighted in most submissions as was the need for *“genuine commitment, partnership and collaboration”* (NPHS, 1b) with Aboriginal organisations when planning multifaceted policy responses. Realising Aboriginal people’s right to self-determination was described as essential to policy success, with one organisation describing it as “ *the foundation of true progress”* (IOE, 5a). Almost all submissions emphasised the need for increased Aboriginal involvement in and, ideally, control of health policy decisions. For example:

“ *Without exception, where Aboriginal people and communities lead, define, design, control and deliver services and programs to their communities, they achieve improved outcomes” *(IOE, 5a).

###  Adoption of Key Issues in Final Policy Documents

Table 4 summarises the extent to which the key issues raised by Aboriginal stakeholders were addressed in consultation committee reports and final policy documents. Committee reports were much more likely to recommend action on the issues raised by Aboriginal organisations compared to final policy documents, which were more likely to defer action or acknowledge the issue without committing to action. For example, committees for the NPHS, WIU and IOE made recommendations to improve food security for Aboriginal communities; however, policy documents deferred to existing strategies in remote communities such as *Outback Stores *and the *Northern Territory Community Stores Licensing Scheme*. Similarly, committees were supportive of food subsidies and other regulatory strategies, but these were not recommended in final policies. Only the NPHS specifically addressed capacity building for Aboriginal health promotion and action on the social determinants of health; social determinants were merely acknowledged or completely absent in other policy documents. Also lacking from policy documents was support for culturally-appropriate primary healthcare programs and services, as endorsed in submissions. Finally, when it came to governance arrangements, policy recommendations generally supported multi-sectoral collaboration but self-determination was not acknowledged or recommend in any of the committee reports or policy documents.

**Table 4:** Uptake of Key Issues Raised in Submissions in Committee Reports and Final Policy Documents

**Key Issues Raised in Submissions**	**NPHS** **Committee**	**NPHS** **Final**	**WIU** **Committee**	**WIU** **Final**	**NFP** **Final**	**ADG** **Final**	**IOE Committee**
**Improve Healthy Food Access and Promotion**							
Food insecurity							
Capacity building for health promotion							
Food subsidies							
Industry regulation							
**Address the Social Determinants of Nutrition**							
Culturally-appropriate interventions/ services							
Inaction on social determinants							
**Strengthen Governance of Public Health Policy**							
Self-determination							
Multi-sectoral collaboration							

**Abbreviations: **NPHS, National Preventative Health Strategy; WIU, Weighing it Up; NFP, National Food Plan; ADG, Australian Dietary Guidelines; IOE, Inquiry into the Obesity Epidemic. Legend: Action recommended. Partially recommended. Action deferred to alternative/existing strategy. Issue acknowledged but no action recommended. No action recommended.

## Discussion

 This is the first analysis of Aboriginal organisational engagement in Australian population-wide food and nutrition policy processes and the extent to which their perspectives are included in final policy documents. Findings suggest that, despite 10 years of advocacy to reduce health inequity and operationalise the right to self-determination, few Aboriginal organisations have participated in food and nutrition policy processes. Furthermore, as far as we could determine from this analysis of publicly available documents, none of the committees governing the consultation processes for the policies under analysis included Aboriginal people. From a political economy perspective, Aboriginal interests and ideas may have been limited by the colonial practice of excluding Aboriginal people from policy development and decision-making institutions of Government. Therefore, our analysis suggests that population-wide food and nutrition policy-making is culturally unsafe by design because it disempowers the self-determination of Aboriginal people. We elaborate on these concepts in light of our findings below.

###  Political Economy (Interests, Ideas, Institutions)

 The concept, ‘interests,’ is concerned with the state of ‘power’ in policy debates.

This includes whose interests are being served through policy-making, how resources are distributed and who stands to gain from this arrangement.^44^ In our analysis, submissions from Aboriginal organisations represented <1% of all written submissions from organisations. Considering Aboriginal peoples comprise 3% of the total population in Australia,^65^ this demonstrates the under-representation of Aboriginal interests in population-wide food and nutrition policy debates. Across the 10-year period analysed and 17 policy processes identified, only 11 of the 1479 submissions made by organisations were from Aboriginal organisations. This may be because, although food and nutrition policy affects Aboriginal people, population-wide nutrition policy is not the core interest of these organisations. Nonetheless, food security and nutrition are key components of the holistic approach to improving health and well-being for Aboriginal people.^31^ The broader prevention scope of the NPHS (beyond nutrition) may explain why this policy process had the highest number of submissions from Aboriginal organisations (n = 5) as it may have been more aligned with the interests of Aboriginal Community Controlled Health Organisations.

Another possible explanation for the small number of Aboriginal submissions is the excessive burden of policy and administrative processes these organisations are required to participate in to remain accredited.^66^ Aboriginal organisations must also divide their time and attention between responding to national, state and territory policy processes regarding both Aboriginal-specific and population-wide issues to ensure their interests are equitably represented. It is possible that, after addressing Aboriginal-specific issues, there is little capacity to participate in other policy processes. In 2019 alone, approximately 20 Australian national population-wide health consultations were open for submissions.^67^ It is unlikely that Aboriginal Community Controlled Health Organisations would have the capacity to respond to every policy consultation process.

In the battle for scarce resources, majority interests can earn political favour more readily.^44^ Aboriginal organisations must compete against the larger, well-resourced and more numerous mainstream organisational and corporate interests. Industry interests frequently undermine public health actors in nutrition policy development.^68,69^ The power of industry interests in watering down public health recommendations in favour of agricultural prosperity was demonstrated during the development of the NFP.^70^ It has been suggested that public health nutrition actors have difficulty generating a cohesive, unified position to strengthen their voice over commercial and political interests.^71^ Our analysis suggests that Aboriginal organisations, as minority actors within public health, have limited power in the food and nutrition policy process to compete with these dominant interests. We suggest that this is not a “full realization…of ensuring participation of Indigenous peoples on issues affecting them” (UNDRIP Article 41). ^22^

###  Ideas

Analysis of ideas seeks to understand how certain perspectives gain prominence in political debates. Transforming ideas into action requires coordinated, strategic communication deployed by actors with shared ideas about a particular issue.^44,72^ Our findings suggest Aboriginal organisations consistently advocated for common ideas in their submissions (eg, food security, cultural safety, self-determination). Despite well-organised actors, deeply embedded cultural orientations can form impenetrable barriers to changing policy development practices.^38^ For Aboriginal organisations, barriers include marginalisation, institutional racism and policy discourse based on deficits.^73^ It has been suggested that governments resist giving greater power to Aboriginal people as this could destabilise entrenched colonial ideas and practices.^74^ Furthermore, Aboriginal peoples’ “ideas” may not fit with “needs” as defined by Western policy-makers.^75^

Aboriginal organisations frequently recommended structural and environmental strategies in their consultation submissions, but policy documents were more likely to recommend behavioural approaches. It is argued that a focus on behavioural deficits in Aboriginal policy is part of the enduring legacy of forced assimilation and ongoing colonisation.^73^ Previous research suggests that solutions focused on behaviour modification are more politically favourable for governments.^71,76,77^ The food industry provides strong opposition to ideas concerning regulatory strategies proposed by public health advocates.^76^ Our findings demonstrate that key ideas raised by Aboriginal organisations during the submission process also rarely penetrate food and nutrition policy. Although it was beyond the scope of this analysis to determine the extent to which the ideas proposed in non-Aboriginal organisations’ submissions influenced policy, the limited progress made by Australian governments in implementing population-wide food policy actions recommended by public health advocates, suggests that this issue is not limited to Aboriginal organisations.^78^

###  Institutions

The findings of this research suggest that the institutional arrangements for participation in national food and nutrition policy processes structurally disadvantage Aboriginal organisations. Aboriginal organisations, like all external stakeholders, participate in policy processes by making submissions and participating in consultation meetings. In many cases, these processes were overseen by a government-appointed standing committee (with no clarity on the selection criteria). This research identified that written submissions were the most common consultation method in food and nutrition policy and these had limited Aboriginal participation. Alternative methods such as round tables, public hearings, public meetings, and workshops were held for some policy processes but were not consistent across the policy episodes. The process of requiring written submissions to participate in policy decisions is a colonial institutional practice that may be inconsistent with Aboriginal oral communication styles where cultural values are infused.^79^ Furthermore, the committees reviewing stakeholder submissions before making recommendations to government are almost entirely comprised of non-Aboriginal people, and the process of writing and editing is opaque. This increases the likelihood that Aboriginal voices will be filtered out in institutional decision-making processes.^80^ Australia’s National Health and Medical Research Council has recently proposed including at least 2 Aboriginal representatives on all future guideline development committees.^81^

According to the United Nations Declaration on the Rights of Indigenous Peoples, to which Australia is a signatory, First Peoples have the right to participate in decision-making regarding all matters that affect them; this includes both Aboriginal-specific and population-wide policy.^22^ However, the findings of this research suggest that, despite increasing engagement and participation of Aboriginal organisations in Aboriginal-specific policy-making, when it comes to population-wide policy processes, Australian governments, irrespective of the political party in power, have been less successful at operationalising the right to self-determination. While Aboriginal-specific policy stresses self-determination and cultural safety as central to policy formulation, population-wide food and nutrition consultation mechanisms exclude the cultural ideas and interests of Aboriginal people.

In contrast to Australia’s absence of a treaty with its First Peoples, the New Zealand treaty of Waitangi binds the New Zealand government to consider Māori rights to self-determination in all policies, enabling Māori to maintain a direct voice to parliament.^82^ The institutionalisation of this domestic human rights instrument is cited as a key factor underpinning the relatively lower levels of Indigenous health inequity in New Zealand compared with Australia.^83^ Unlike New Zealand, Australia does not have dedicated seats in its national parliament for representation of First Peoples. In fact, the first (and only) 2 Aboriginal members of the Australian House of Representatives were elected during the period under analysis, in 2010 and 2016.^84^ The Uluru Statement from the Heart is a consensus statement from Aboriginal Australia which advocates for a constitutionally-enshrined voice to Parliament so that Aboriginal people can have a consistent voice on all policy decisions that affect them.^58^ This research provides further evidence for the need for an Aboriginal voice to be embedded within policy-making institutions in Australia.

###  Cultural Safety

It is through Aboriginal participation that cultural values, needs and preferences for action can be reflected in policy development processes through to strategic documents and government actions. Our research reveals the exclusion of Aboriginal people from the committees overseeing the development of food and nutrition policy actions and the marginalisation of Aboriginal interests and ideas in policy documents. Māori scholar, Dominic O’Sullivan argues that the democratic expectations of First Nations peoples are conditioned by colonial experiences.^83^ Came et al found in New Zealand that, despite the Treaty of Waitangi, the knowledge and interests of Māori and Pasifica leaders were devalued in policy participation processes.^85^ Our analysis of Australian policy processes found that arguments about social determinants of health, cultural appropriateness and self-determination were excluded from the policy agenda. The complete absence of Aboriginal people from population-wide national food and nutrition committees over a 10 year policy trajectory affirms the Australian norm of ‘legal invisibility’ of Aboriginal people in Australian policy-making, and recreates institutional inequity in policy responses.^54^

We found that the vast majority of submissions in population-wide food and nutrition policy processes were from non-Aboriginal organisations. Along with the membership of oversight committees consisting of non-Aboriginal experts, civil servants and politicians, and the control of the policy process by governments, politicians and government statutory organisations, this indicates the dominance of non-Aboriginal voices in the legitimation of knowledge, decisions about who makes the rules and where resources are allocated. This institutionalised attitude runs contrary to establishing participatory systems and processes through which Aboriginal people can express self-determination.^22^ An attitude that diminishes or disempowers Aboriginal cultural identity can create an environment that is alien, culturally unsafe and a dangerous place for First Nations peoples to be.^48,86^ Our findings demonstrate the need for critical reflexivity in the institutional design of policy development processes so that they are culturally inclusive and address power imbalances and, thereby, enable genuine participation of Aboriginal peoples and culturally safe food and nutrition systems.

###  Limitations

This research has several limitations. First, it is possible that some Aboriginal policy submissions were missed if the organisation requested that it not be made publicly available. Aboriginal organisations may have also contributed through other consultation mechanisms such as roundtables public meetings and informal networks^87^; however, these were not included in the current analysis as it was difficult to capture who attended these meetings. Furthermore, we focused on formal mechanisms of participation and acknowledge the influence of informal networks of influence through which Aboriginal peoples influence heath policy. Thus, we cannot be certain that we have captured all of the Aboriginal perspectives presented in food and nutrition policy debates. Despite this limitation, our findings indicate that participation of Aboriginal organisations in national population-wide food and nutrition policy processes has been limited.

 Another limitation is that we were unable to attribute the actions recommended in policy documents exclusively to the submissions made by Aboriginal organisations. It is possible that non-Aboriginal organisations were also advocating for similar issues such as food security and regulatory policies. The submissions made by other organisations have not been examined for comparison. Additionally, the inclusion of recommendations in policy documents does not guarantee that these will be implemented in practice. Future research should examine the full range of stakeholder perspectives and the extent to which policy recommendations are implemented by governments.

The focus of this analysis was national-level, population-wide food and nutrition policy. This does not capture the full extent of Aboriginal participation in food and nutrition policy-making processes in the Australian context. State and Territory governments develop their own health policies, including food and nutrition; however, analysis of the degree to which Aboriginal people have participated in these policy processes was beyond the scope of the current study. Furthermore, Aboriginal and Torres Strait Islander health is a discrete area of national health policy, which operates in parallel with population-wide food and nutrition policy processes.^37^ Our population-wide focus meant that we did not analyse participation in food and nutrition policies targeting Aboriginal people, where engagement would likely be greater.

## Conclusion

This research has demonstrated that over a 10-year period in Australia: (*i*) very few Aboriginal organisations have participated in national, population-wide food and nutrition policy processes; (*ii*) common and consistent themes in Aboriginal organisations’ policy submissions included food insecurity, culturally-appropriate programs, multi-sectoral collaboration, capacity building, social determinants, and Aboriginal peoples’ right to self-determination; and, (*iii*) Aboriginal recommendations were seldom addressed in final policy documents. These findings indicate existing population-wide food and nutrition policy processes are culturally unsafe and systematically disadvantage Aboriginal Australians. With regard to population-wide food and nutrition policy, Australia is not meeting its obligations under the United National Declaration on the Rights of Indigenous Peoples. Our analysis could be replicated across other public policy areas in order to further evaluate the implementation of this human rights framework. Alternative policy-making mechanisms are required to adequately address Aboriginal interests and ideas and enable self-determination in the area of food and nutrition. The Uluru Statement from the Heart provides a moral and human rights imperative for improving governance arrangements for Australia’s First Peoples.

## Acknowledgements

 We respectfully acknowledge the Aboriginal organisations who participated in the policy processes included in this analysis. This research took place on the lands of the Wurundjeri, Wathaurong, and Awabakal peoples of Australia. ML is a Ngiyampaa man.

## Ethical issues

 As this research only involved analysis of publicly available, organisational documents institutional ethics approval was not required.

## Competing interests

 Authors declare that they have no competing interests.

## Authors’ contributions

 JB conceived of and designed the study with support from KB. MG carried out the search and undertook the political economy analysis with support from JB. ML undertook the cultural safety analysis. All authors discussed the results and contributed to the preparation of the manuscript.

## Funding

 JB is supported by a an Alfred Deakin Postdoctoral Research Fellowship and a VicHealth Research Impact Grant. KB is supported by a Heart Foundation Future Leader Fellowship (102047).

## Authors’ affiliations

^1^Global Obesity Centre, Institute for Health Transformation, Deakin University, Geelong, VIC, Australia. ^2^School of Health and Social Development, Deakin University, Geelong, VIC, Australia. ^3^Committix Pty Ltd., Newcastle, NSW, Australia.
